# A Low-Cost Indoor Activity Monitoring System for Detecting Frailty in Older Adults

**DOI:** 10.3390/s19030452

**Published:** 2019-01-22

**Authors:** Thomas Tegou, Ilias Kalamaras, Markos Tsipouras, Nikolaos Giannakeas, Kostantinos Votis, Dimitrios Tzovaras

**Affiliations:** Information Technologies Institute, Centre for Research and Technology Hellas, Thessaloniki 57001, Greece; kalamar@iti.gr (I.K.); tsipouras@iti.gr (M.T.); giannakeas@iti.gr (N.G.); kvotis@iti.gr (K.V.); dimitrios.tzovaras@iti.gr (D.T.)

**Keywords:** indoor localization, Bluetooth beacons, room-level accuracy, frailty level assessment

## Abstract

Indoor localization systems have already wide applications mainly for providing localized information and directions. The majority of them focus on commercial applications providing information such us advertisements, guidance and asset tracking. Medical oriented localization systems are uncommon. Given the fact that an individual’s indoor movements can be indicative of his/her clinical status, in this paper we present a low-cost indoor localization system with room-level accuracy used to assess the frailty of older people. We focused on designing a system with easy installation and low cost to be used by non technical staff. The system was installed in older people houses in order to collect data about their indoor localization habits. The collected data were examined in combination with their frailty status, showing a correlation between them. The indoor localization system is based on the processing of Received Signal Strength Indicator (RSSI) measurements by a tracking device, from Bluetooth Beacons, using a fingerprint-based procedure. The system has been tested in realistic settings achieving accuracy above 93% in room estimation. The proposed system was used in 271 houses collecting data for 1–7-day sessions. The evaluation of the collected data using ten-fold cross-validation showed an accuracy of 83% in the classification of a monitored person regarding his/her frailty status (Frail, Pre-frail, Non-frail).

## 1. Introduction

While GPS offers an adequate solution for outdoor localization, indoor localization is still an open challenge. There are many algorithmic and hardware approaches, depending on the desired accuracy and cost. The available localization systems provide position-related information (e.g., in museums), advertisements (e.g., in shopping centres) and guidance in large indoor places (e.g., airports). These systems are also used for asset tracking in industrial environments. The majority of the available solutions are costly and complex to install, focusing on commercial purposes. Few relevant localization applications are oriented to medical purposes. However, recent advances in IoT (Internet-of-Things) applications have shown that they can be invaluable for health monitoring. We tried to create a new perspective to indoor localization systems and exploit a new potential of them, i.e., the correlation of indoor localization data with the medical status of individuals and specifically their frailty status.

Frailty is a condition of increased vulnerability and decreased endurance appearing in an ageing population, caused by a cumulative decline in the physiologic systems of an individual, resulting in increased risk of disability, morbidity, psychological decline, hospitalization or death. The increased vulnerability of frail older persons to stressors and health implications also has an impact on effective healthcare planning and delivery by healthcare organizations. Early detection of frailty indicators in the individual’s health status can assist in preventing or delaying frailty symptoms, or even reversing the process [1].

Assessment of frailty is often based on quantitative phenotypical criteria, such as unintentional weight loss, exhaustion, weakness, slow walking speed and low physical activity, as used in the widely used Fried frailty index [2]. These criteria are mostly gathered through questionnaires or interviews with the individuals, during clinical sessions or visits of clinical personnel to the older person’s premises, thus they are usually considered as impractical for continuous care. Unobtrusive and real-time measurement of health status through wearable or ambient sensors can significantly assist in the collection of more comprehensive data and the early detection of adverse outcomes. Activity monitoring can prove valuable for assessing frailty, since it is directly relevant to frailty indicators, while at the same time it can be performed constantly, in a manner that is unobtrusive for the older person.

In this paper, a low-cost indoor localization system is proposed for monitoring the mobility behaviour of older individuals and a study is performed to assess the correlation between measured indoor activities of an older person and his/her frailty status, as assessed by well-known frailty criteria. The localization system was first presented in [3], which is hereby extended with the addition of the frailty assessment study. Preliminary results of this study, with a small dataset, were presented in [4]. The measurements of activity are collected by monitoring the position of the older person in the house, at room level. For our purposes it was considered more meaningful to know the position of the monitored person in the terms of logical areas, such as rooms and corridors, rather than the precise locations specified by coordinate information. The proposed system was designed giving priority to ease of installation, in order to collect sufficient data from several persons. The purpose of focusing on easy setup was that non-technical staff such as nurses can install the system following simple and short instructions, without needing additional details such as distances between the beacons or the floorplan of each house. The hardware components of the system include Bluetooth beacons positioned in the localization area and a tracking device (e.g., a smartphone) carried by the individuals. In a second phase, the collected data were examined to extract correlations between the indoor localization habits and the frailty status of the individuals. Several features of activity are extracted from the raw measurements, related to room transitions and to how long the person spends in a room, and are assessed in terms of their correlation to the frailty status. The features are then used to train frailty status predictors, and to evaluate the accuracy of predicting the person’s frailty status using the measured activity data.

The proposed indoor localization system was firstly presented in [3] and preliminary results regarding its frailty assessment capability were presented in The rest of the paper is organized as follows: Section 2 includes related work about indoor localization and frailty monitoring, Section 3 presents the proposed localization system. Section 4 includes an evaluation of the system regarding its accuracy in room estimation and its frailty assessment capability. Finally, the discussion and the conclusion present a summary and directions/considerations for future work.

## 2. Related Work

The following section presents localization and frailty assessment methods.

### 2.1. Localization

Indoor localization is usually handled using RF (Radio Frequency) signals and protocols, such as Bluetooth, WiFi, RFID, UWB (Ultra-Wide Band) and ultrasound [5,6]. Bluetooth beacon-based solutions have especially gained attention recently, due to their relative low cost and minimal requirements in signal receivers, since widely used smartphones or smartwatches can readily be used as such. Beacon-based systems are effectively used in commercial applications, such as navigating users in shopping centers, museums, airports, etc., with their main purpose being offering location-related advertisements. However, user localization in such systems often requires the exact floorplan of a facility, a large number of installed beacons, or large training datasets, which increase the total installation cost [7]. Medical applications can benefit from beacon-based localization. However, in order to achieve wide applicability, low-cost solutions, with minimal training need to be employed.

Theoretically, there are two main categories of beacon-based localization methods: the ones based on trilateration/triangulation and the ones based on fingerprinting. *Trilateration* is the process of determining the location of an individual by measuring its distance to at least three fixed points of known positions, such as the positions of three beacons. The position of the individual is computed by solving a least-squares problem [8,9]. In case of Bluetooth beacons, distance is usually measured in terms of the RSS (Received Signal Strength) value of each beacon, as measured from a receiver (e.g., smartphone) at the position to be estimated. Current trilateation-based methods focus on increasing the accuracy and efficiency of solving the main least-squares problem, through incorporating long distance shadowing [10], employing multiple iterations [9,11] or utilizing ring-shaped overlapping regions [12]. *Triangulation* methods, instead of being based on measuring the distance from fixed points, work by computing angles from reference points [8], based on the time-of-arrival (TOA), the time-difference-of-arrival (TDOA), or the roundtrip-time-of-flight (RTOF) of the radio signals. Triangulation is being used for GPS positioning; however, their application for indoor localization is limited, due to the low capability of smartphones to perform the above types of measurements.

**Figure 1:**
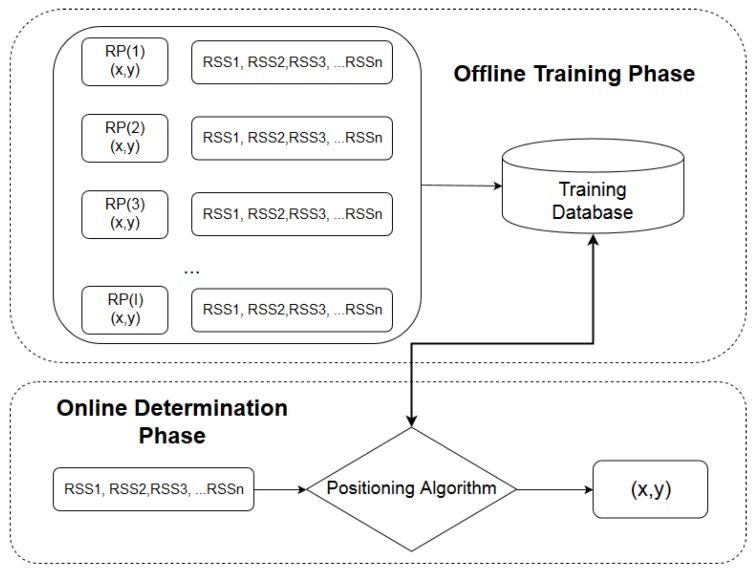
A diagram of the fingerprinting method.

A different concept is adopted by *fingerprinting* methods. These methods are based on collecting off-line RSS measurements (fingerprints) from fixed beacons at multiple reference points in the indoor environment. Later, during the on-line localization phase, the current RSS measurements of the tracked device from the fixed beacons are compared to the collected database of fingerprints and the current location is estimated by considering the closest fingerprint matches, as seen in Figure 1 [13,14]. Classification techniques are utilized in order to perform fingerprint matching, such as *k*-nearest neighbors (kNN), support vector machines (SVM), Naïve Bayes [15] and neural networks [16]. Fingerprinting methods have the advantage of not requiring the knowledge of the positions of the fixed beacons. However, for exact localization, a large number of reference training points needs to be collected. Apart from the above broad categories, other methods include fusing measurements from multiple sensors available in smart devices, such as magnetometers, accelerometers, gyroscopes and ambient light sensors, to perform localization [17]. Measurement fusion is often accomplished through Kalman filters [18].

Regarding room-level accuracy localization, in [19] a beacon threshold-based method is presented, where the dimensions of the rooms are used in addition to RSSI measurements in order to improve accuracy. The proposed method uses the dimensions of the room in order to create thresholds for the RSSI measurements defining if someone is inside or outside of a room. It is not a fingerprint-based method and shows an improvement in the accuracy compared to the situation where only the highest RSSI measurements were used to estimate the room where a person is located. The disadvantage of this method is that the dimensions of each room are needed; moreover the beacons should be placed at the ceiling of each room, making difficult the installation procedure of such a system in multiple houses. In [20] an In-Room presence detection system is presented. The system uses beacons in the doors in order to recognize when a person enters the room. Specifically, one beacon with motion sensor is placed at the door of a room and the other beacon is placed inside the room close to the entrance. In case of door motion, the system starts to record RSSI measurements from the two beacons, while the user is carrying the smartphone. Examining the differences between the two recorded RSSI time-series, the method is able to recognize when someone enters or leaves the specific room. The proposed system achieves high accuracy, near 99%; however, it assumes that each door is always closed when someone wants to enter or leave a room. In addition, motion sensor beacons and a minimum requirement of two beacons per room are needed, significantly increasing the total cost. The proposed system is more suitable for large indoor environments, such as workplaces, event attendance monitoring, etc. and not for houses, where the doors between rooms remain open for most of the time. In [21,22], a Bluetooth fingerprint-based room localization system is presented, where additional signal features are used among the RSSI measurements, such as the Link Quality (LQ) and the Cellular Signal Quality (CSQ), using a combination of Bayesian statistics and Support Vector Machines for classification. Moreover, the proposed combination in [22] is used in order to improve the accuracy of [21]. In our case, such additional measurements need specific hardware and cannot be extracted from BLE Bluetooth signals. Table 1 presents a summarized comparison of the previously mentioned room-localization systems with the proposed system. We compared the systems based on the ease of installation, the accuracy, the cost/hardware availability, which refers to the easiness of finding and purchasing the specific hardware, and the system’s suitability for house environments.

Localization-collected data are used in correlation with other health-related data in e-health platforms. In [23], a medical platform focused on people using wheelchairs is presented. The platform introduces new hardware approaches such as the smart wheelchair with localization and falling sense abilities. The platform monitors, among others, the heart rate or the ECG time-series. Moreover the platform presents a novel social network to improve the efficiency of resource sharing. The FrailSafe project [24] focuses on better understanding frailty and its relation with other health conditions and indicators. It provides cloud platform functionalities for the monitored person, the doctor and the relatives. Frailsafe monitors older people’s health condition variables, indoor, outdoor and social media activity. The collected data are used in order to find new non-intrusive ways for assessing older people’s frailty and health status such as tablet games. Moreover, it provides automatic suggestions and interventions for individuals. The ACTIVAGE project [25] is an IoT cloud platform-based project, where daily behavioral activity monitoring of older people is performed, using door and motion sensors. Health indicators such as blood pressure or glucose levels are monitored as well. Alarms and notifications are provided in urgent situations. ACTIVAGE focuses on supporting and extending the independent living of older adults in their living environments, and responding to real needs of caregivers, service providers and public authorities.

### 2.2. Frailty Monitoring

Activity-related information, such as monitoring indoor movements, are closely related to frailty evaluation of individuals. Although frailty is often assessed through metrics of cognition, function, social health, medication, co-morbidities, etc. [26,27,28], common frailty evaluation measures, such as the Fried index [2], are also based on physical activity and mobility to determine the frailty level. Activity-related measures, such as the IADL (Instrumental Activities of Daily Living), have been used effectively for frailty assessment [29]. However, this type of information has mostly been collected through clinical evaluations performed by clinical personnel, which has disadvantages, such as infrequent data collection and increased cost.

Automatic real-time monitoring of the older person’s activity can significantly assist in frailty assessment through continuous monitoring of older people without the need of clinical personnel presence. Monitoring methods have utilized wearable systems [30] and gait speed recognition [31] for activity recognition and frailty risk assessment. Recent works have used motion identification through accelerometers and gyroscopes [32], as well as extraction of activity patterns through ambient sensors for unobtrusive detection of unusual activity [33,34], holistic frailty assessment [35] and rehabilitation [36]. Mobiliy patterns of the individuals have been usually extracted through motion sensors [33,34]; however, localization-based methods such as the ones mentioned in Section II present a promising alternative of lower cost. The discriminating ability of activity and mobility information to detect frailty levels has been assessed through recent studies [37,38,39].

## 3. Proposed Localization System

### 3.1. Architecture

The focus of the proposed localization system is to achieve a balanced trade-off between accuracy and ease of installation. We designed a system with a user-friendly setup procedure that could be used by non-technical staff, in order to collect localization data from multiple persons. Taking into account the limitations regarding cost, user-friendliness and cloud integration, we selected to use beacons and smart-phones, as hardware components of our system. This hardware selection was made due to its availability, its low cost and robustness. Using a smartphone as a tracking device, a GUI (Graphical User Interface) could be developed, facilitating the setup procedure of the beacons from non-technical staff. In addition BLE Bluetooth functionality is available in every budget smartphone in the market, thus cheap smartphones can be used as well. Finally, using a smartphone it is possible to integrate cloud functionality, which is necessary to collect data from multiple users in a long-basis. A smartwatch could also be used as a tracking device. In our approach we kept a low hardware cost and a small number of beacons to be used in NLOS (Non Line Of Sight) environments.

The designed localization system is based on the process of Received Signal Strength (RSS) measurements, broadcasted by beacons, placed in the localization area of each monitored house. The beacons are small passive Bluetooth Low Energy (https://kontakt.io/beacon-basics/what-is-a-beacon) devices which broadcast beEnergytween small time intervals information messages, containing among other fields such as their ID. These messages are scanned continuously by the smartphone, which measures the Received Signal Strength (RSS) from them. The RSS is compared to a set of RSS values (fingerprints) collected during the setup phase, in order to determine the tracking device’s position. The use of RSS fingerprints has the advantage that it bypasses the need of transforming the RSS values to distance which is highly dependent from the topology of each house and requires a long procedure of taking RSS measurements in various distances from the beacons.

**Figure 2:**
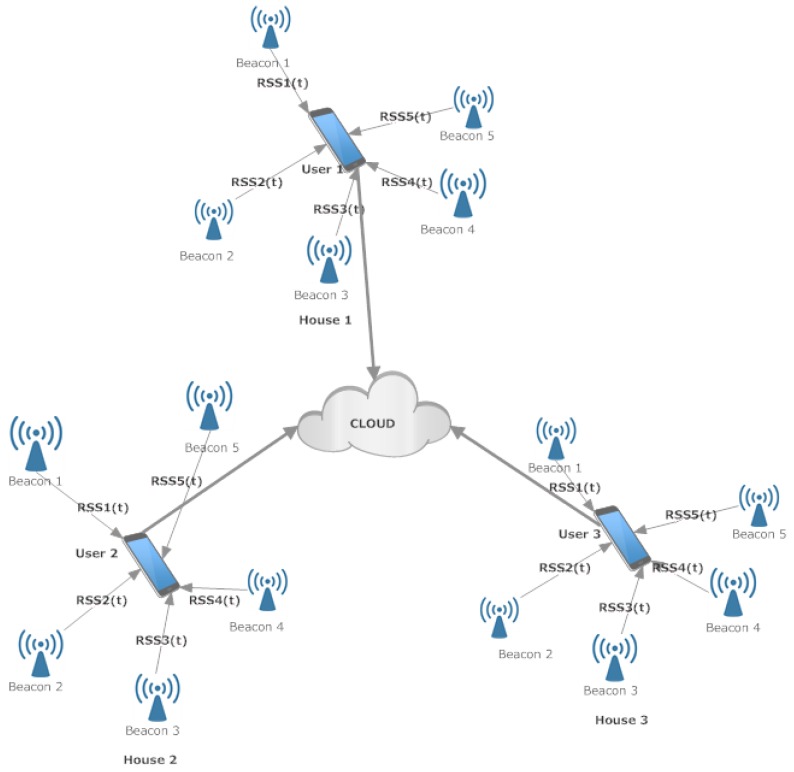
An overview of the system architecture.

The software components of the localization system include an application for setting up the localization installation and collecting RSS fingerprints (installed in a smartphone to be used by healthcare personnel), an application for real-time localization (installed in a smartphone or smartwatch, to be carried by the tracked person), and a cloud service used to collect the data from multiple users of the application. The main features of the system can be seen in Figure 2.

The localization system collects information about the indoor position of the tracked person with room-level accuracy. The main reasons that the system is based on the specific hardware choices are the low cost and the availability of the involved devices (beacons, smartphones, smartwatches). Moreover, using a smartphone for the installation of the setup application allows developing an interface which is necessary in order to collect training data by non-technical personnel (e.g., nurses) with a short procedure, as described in the following sections. Finally, it is quite easy for someone to carry a smartphone in his/her pocket, used as tracking device. The reason that room-level accuracy was chosen is the simplicity of the setup procedure. A different approach with exact coordinate-level accuracy would need a much more complicated setup procedure including floorplans of the houses, distance measurements, and a larger number of beacons, increasing the total cost and the complexity.

The implementation of our approach consists of two phases. The first one is the off-line phase (setup procedure), for collecting training data to be used by the second one, the online phase (indoor localization). The person who undertakes the first procedure is a nurse, the procedure is executed once and it takes only a couple of minutes (2–5 min) in order to be completed. During the online phase, the only requirement is simply the smartphone to be carried be the monitored person in his/her pocket.The collected data are being synchronised with the cloud on a regular basis.

### 3.2. Beacon Setup Procedure

**Figure 3:**
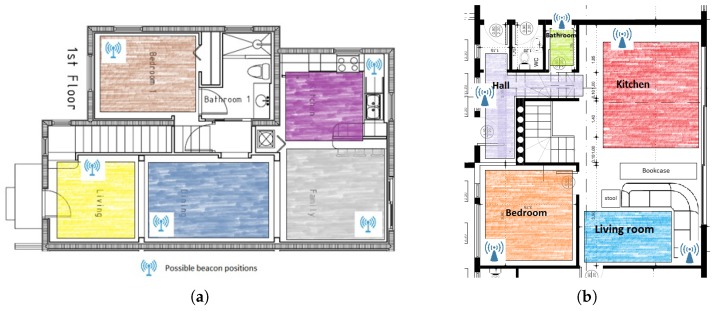
(**a**): Example of a beacon topology in a house. (**b**): Example of a beacon topology in the KRIPIS Smart Home [40] at CERTH/ITI, were the system was evaluated. With different colors are the areas where the user, performing beacon setup procedure, should walk for half a minute in order to create the signal fingerprint for each room.

During the Beacon setup procedure the beacons should be placed in the rooms of the house, in appropriate positions so that they are at least 2 m from each other, in order to collect training data for each room afterwards. The reason for keeping this distance restriction between the beacons is that the collected RSS fingerprints can sufficiently discriminate the rooms. The specific distance was pointed out as a minimum distance between beacons in the setup instructions, which imposes some limitations in the number of beacons to be placed in a room. One beacon per room was used in each house as a good trade-off between accuracy and total cost. Examples of beacon topologies are presented in Figure 3a,b. The beacon positions are denoted with the antenna symbols.

After placing the beacons in appropriate positions in the rooms of the house, the last stage of the off-line phase includes the collection of training data from the RSS measurements from each room. An RSS fingerprint of each room should be created. To perform this stage, the nurse walks in each room for half a minute. The color-shaded areas in Figure 3a,b refer to representative areas inside which the person performing the installation needs to walk.

The creation of the signal fingerprint of each room is a 30-s procedure. Initial tests were performed using 60 s training for each room. Insignificant changes in localization accuracy (<2%) were found when the training time was reduced to 30 s. The selected value was selected as a good trade-off between localization accuracy and total time required for setup, given the fact that non-technical staff undertake this procedure. In each second, different measurements from each beacon are stored. During this short period, the person performing the beacon setup is moving in the area of the specific room. Finally, an average is computed for each beacon representing the RSS fingerprint of the specific room. Formally, the fingerprint for a room is considered as a vector

(1) $$f_{i}=\left({\mathrm{rss}}_{i,1},{\mathrm{rss}}_{i,2},\ldots ,{\mathrm{rss}}_{i,M}\right)\in R^{M},$$

where *M* is the number of the beacons used in a house, and ${\mathrm{rss}}_{i,j}$ is the average from 30 measurements for beacon *j* computed as

(2) $${\mathrm{rssi}}_{i,j}=\frac{\sum_{k=1}^{30}rss_{k}}{30},k=1...30$$

where *k* represents the time moment. The collected fingerprints from all rooms are stored in a file, in order to later be used by the indoor localization application. In order to check whether the training procedure is performed correctly, a step detector is used as a primitive way to validate the training procedure. During the fingerprint collection, in case there is no step detection for one or more 5-s-periods within 30 s, the collected measurements are considered unreliable and the user should repeat the procedure for the specific room. The step detector uses the measurements from the accelerometer of the smartphone and a step is detected each time a certain threshold is exceeded. The specific feature could be improved using the magnetometer of the smartphone as a compass in order to ensure that a user will walk in the whole area of the room. The reason for not using a magnetometer was that not all smartphones have this specific feature.

### 3.3. Indoor Localization Phase

**Figure 4:**
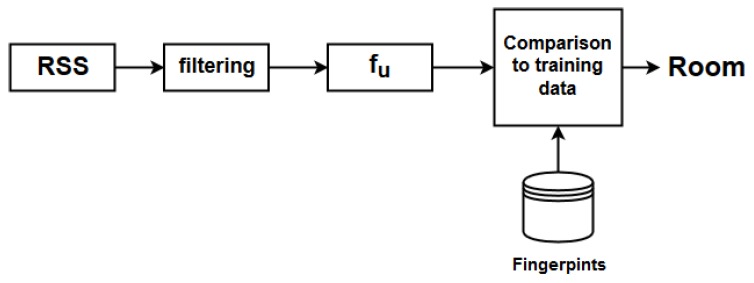
Block diagram describing the room estimation procedure.

After completing the setup procedure, the system is able to perform localization. In general the localization phase is based on the continuous comparison of the current RSS measurements from the beacons with the training data collected during the setup procedure. A block diagram of the smartphone functions running during the localization phase is presented in Figure 4. The output of the process of the online phase is to store locally the room change and the timestamp of this change.

For each beacon, there is a constant length time sliding window with RSS measurements. Each time there is a new measurement from the *j*-th beacon a new value is inserted while the oldest one is discarded. The average and the standard deviation (*s*) are computed for each time window. Values with absolute distance greater than 2×s are discarded and a final filtered average is computed. Having filtered the raw RSS measurements, a vector is formed with the filtered values. This vector is used for the comparison with the training data, the classification and the room estimation. Let

(3) $$f_{u}=\left({\mathrm{rss}}_{1},{\mathrm{rss}}_{2},\ldots ,{\mathrm{rss}}_{M}\right)\in R^{M},$$

be the vector with the filtered RSS values. The Euclidean distance between this current fingerprint and each fingerprint of the database is computed and the minimum distance corresponds to the room where the user of the smartphone is located:

(4) $$\mathrm{room}=\arg\min_{\begin{array}{c} i\in rooms \end{array}}\left|\right|f_{u}-f_{i}\left|\right|$$

**Figure 5:**
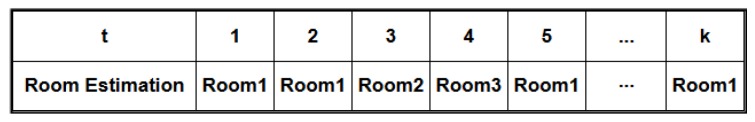
Each time moment the algorithm estimates the possible room.

In addition, in order to decrease fluctuations between rooms, another time sliding window is created with the estimated rooms of the above procedure. Each time moment a new estimation of the room is created and stored in this sliding window while the oldest estimation is discarded as seen in Figure 5. The appearance frequency of each room in this window is computed. Let $fr_{i}$ be the appearance frequency of room *i* in this time window. The final room estimation is:

(5) $${\mathrm{room}}_{\mathrm{final}}=\arg\max\left(fr_{1},fr_{2},\ldots ,fr_{M}\right)$$

when the maximum frequency is above the 80% of the length of the time window, which is 800 time time instances, then the application considers that the user is located in this room. A new time instant is considered when the algorithm has a new RSSI measurement from any beacon. There is also a functionality for estimating when the user is outside the monitored indoor environment. When the smartphone does not receive broadcasts from the beacons used in the training phase for a time period of 4 second, the application considers that the user is outside the house.

**Figure 6:**
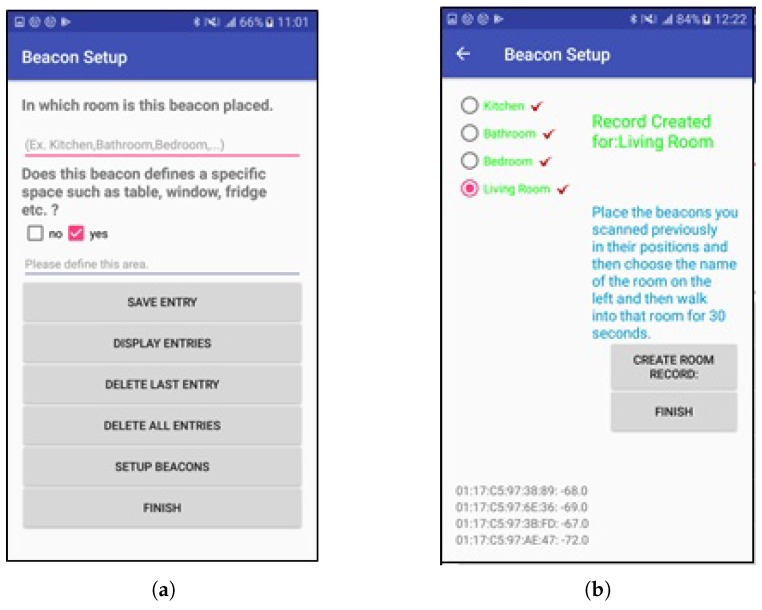
Screenshots from the Beacon Setup application (**a**): The user enters the names of the rooms one by one. (**b**): On the left there is a list of the rooms. Each time the user of the application chooses a room and performs a half minute walk in the specific room.

The above stages of the system are implemented with two applications; namely, the Beacon Setup and the Indoor Localization app. In Figure 6a,b relevant screenshots from the Beacon Setup app are presented, where the medical staff enters the names of the rooms one by one Figure 6a and then walks in the area of each room for 30 s Figure 6b. The screenshots are indicative of the ease of the setup procedure.

The central cloud repository is being synchronized for each user periodically, by transmitting batches of locally stored data. The data contain the user ID, room transitions and the relevant timestamps. The data transmission can occur automatically too, transmitting each room transition and the timestamp when this happens.

## 4. Evaluation of the Proposed System

Two kinds of evaluations were performed. The first one regarding the localization accuracy and the second one regarding the accuracy for frailty classification.

### 4.1. Evaluation of Indoor Localization Accuracy

The accuracy of the proposed localization system was tested in the KRIPIS Smart Home [40] at CERTH/ITI, Thessaloniki, Greece. The installation included five beacons in five rooms of the house. The area of the house is 8.6 m × 9.35 m, equal to 80.41 m^2^. Another additional information about the specific floorplan is that there is no wall between the kitchen and the living room. The reason for mentioning that is that a wall is a source of additional attenuation in RSS measurements [41]. The focus of the tests was to evaluate the accuracy of the system in realistic conditions, where other sources of interference coexists, such as WiFi routers and other persons except for the monitored one. In addition, the application was tested while a user, carrying the tracking device, was standing in four different directions in each position. In general, the propagation of the signal changes when one or more obstacles interfere between the Line of Sight of a transmitter (beacon) and a receiver (smartphone). In certain directions the human body blocks completely these LOS conditions and acts as a significant source for signal attenuation among other obstacles. The user remained in each position for four minutes, one minute for each direction. A smartphone was used for the test, for running both the setup and the localization applications. During the evaluation, the phone was in the user’s pocket. A LG Nexus 5x smartphone and Sensoro beacons were used. The Sensoro beacons were chosen due to their low price and their long lasting battery (they use 4 AA batteries). In general, the RSSI measurements differ when using different smartphone devices. In our system, in each monitored person the same LG smartphone was used both for training and localization, which eliminates inaccuracies due to different hardware selection. In addition each monitored person used the same smartphone model. In general the best practice is to use the same phone for training and localization.

**Figure 7:**
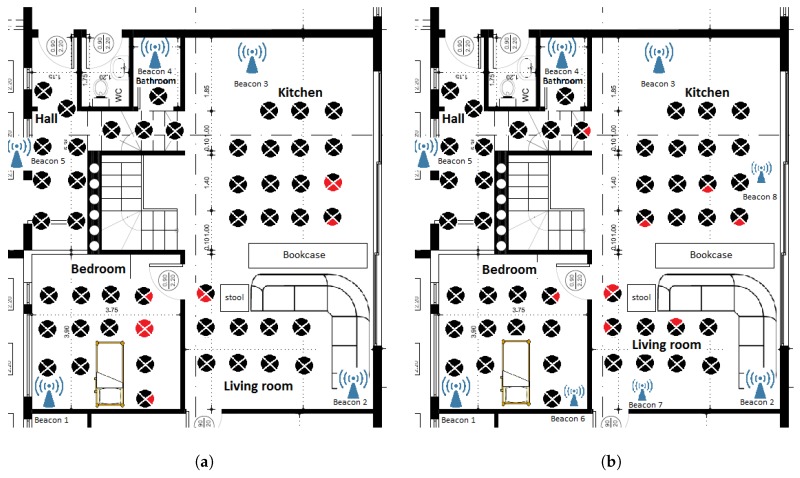
The results from the 2 conducted tests. (**a**) The floorplan of the house where the evaluation test was conducted. Each section of the circle represents a different direction of the user. Red color represents wrong room estimations; (**b**) The results of the second experiment where the number of beacons increased from five to eight. Each section of the circle represents a different direction of the user. Red color represents wrong room estimations.

In Figure 7a, the results of the test are presented. The total points were 48. Each circular sector corresponds to a specific orientation of the user, black color refers to a successful room estimation, while a red one to a false estimation. The test was conducted while the user was standing in four different directions in each point. The reason for testing the algorithm in multiple directions is that the human body attenuates the Bluetooth signal, as seen in [42,43]. If the human body interferes between a smartphone and a beacon then additional attenuation is inserted in the RSS measurements. Considering each direction as a different position the total positions are 48×4=192. The total number of wrong estimations are 12, which corresponds to a percentage of 93.75% in succesfull room estimation.

Another experiment was conducted increasing the number of beacons from five to eight. Two beacons were placed in the three largest rooms of the house instead of one, dividing each of these rooms into two subspaces. The training phase was conducted considering each subspace as an individual room. The increase of beacons increased the room estimation accuracy from 93.75% to 95.31%. The results of the second testbed are presented in Figure 7b.

The localization system was evaluated in an environment where sources of interference including Bluetooth, BLE Bluetooth, WiFi and ZigBee signals, as well as human body interferences, coexisted. In [44], an extended study is presented regarding coexistence and interference tests between the previously mentioned factors. The coexistence of BLE Bluetooth and WiFi shows an increase in RSSI values from 1 dBm to 5 dBm when WiFi interferes to BLE Bluetooth transmitted packets, depending on the distance of the source of the interference. On the other hand, BLE Bluetooth coexisting with Bluetooth and ZigBee devices does not have an impact in the measured RSSI values. In [44], the magnitude of the human body interference is also measured using pig skin to cover the receiver. A drop between 1 and 6 dBm was found in NLOS conditions. Our system despite the sources of interference achieved sufficient accuracy in room estimation, probably due to the distance of the WiFi source of interference from the localization area, namely the WiFi router is outside the localization area. As for the human body interference it is not so intense as presented and measured in [44], where the sensor tag is covered by pig skin, on all sides so the RSSI drop is smaller than in [44]. Finally, the filtering procedure of the RSSI measurements contributes to the increase of total accuracy. An examination of the effects of the interferences in the system’s accuracy will be considered in future experiments.

### 4.2. Evaluation of Frailty Monitoring Capability

In order to evaluate the capability of the proposed system in frailty monitoring, a large-scale trial was conducted. The trial included 271 subjects (102 males and 169 females), in three different locations (Patras–Greece, Nicosia–Cyprus, and Nancy–France). The recording data include the subject’s ID, frailty status, location, birth year, gender. The frailty status is defined as non-frail, pre-frail and frail. The number of subjects in each frailty status is depicted in Table 2. The dataset included subjects with ages of 76.8 ± 5.2-year-old for males and 76.7 ± 5.4-year-old for females.

The indoor localization system was installed in the house of each subject for a number of consecutive days (1–7). After the installation, each subject was instructed to carry the mobile phone while moving around the house and performing daily activities. The mobile phone measures the RSSI values from the beacons of the house continuously; each time the subject exits a room and enters another room, a transition is recorded. This transition includes the label of the room that the subject entered and the timestamp that this change occurred. A record of room transitions is generated from each subject while moving around the house, performing his/her regular activities. A room transition record, contains the following fields: (i) the userID, which is a unique ID of the subject, (ii) the label of the room that the subject walked into (the labels of the rooms have been registered during the installation process of the hardware by the clinical personnel and they are unique in each house), and (iii) the timestamp of the transition. Thus, each row in the room-transition record corresponds to a room change.

**Figure 8:**
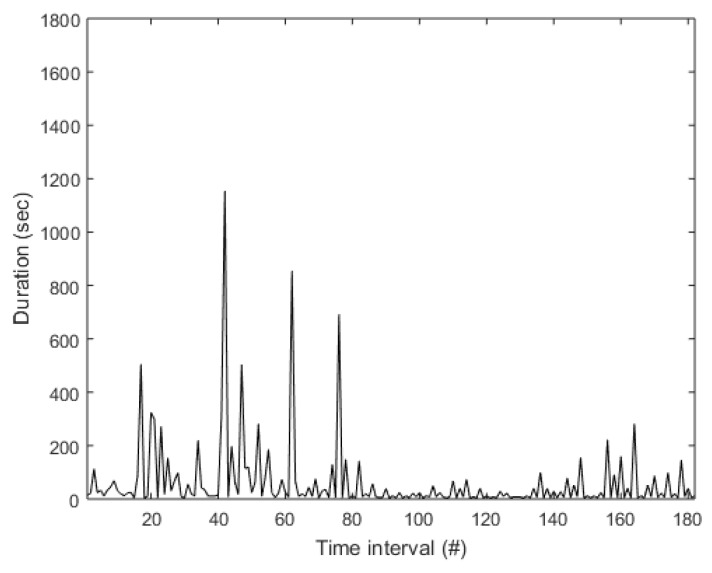
Example of the time-intervals signal.

The room-transition records were processed in order to extract the time-intervals signal, which is the signal recording the time interval that the subject remained in each room. For this purpose, the interval between successive transitions is calculated, and the sequence of all time-intervals in each room-transition recorded is extracted. An example of the time-intervals signal is presented in Figure 8.

**Figure 9:**
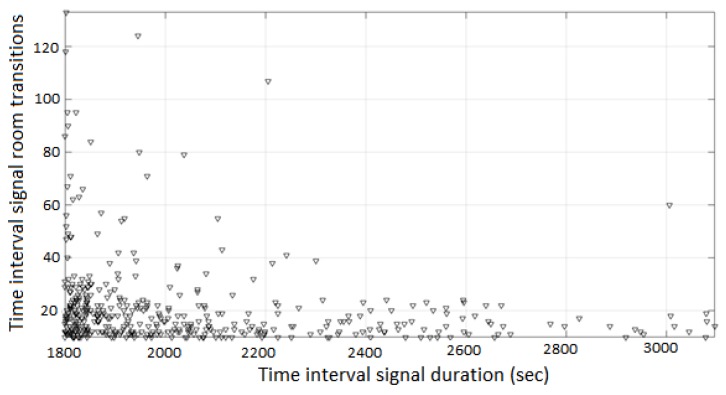
Signal duration (s) vs. number of room transitions, in the time-interval segments.

A segmentation process was applied, generating time-interval signals with duration ≥1800 s. The process was based on the running sum of the time intervals in the signal, generating a segment when the running sum exceeds the time threshold; the actual length of each segment is determined from the length of the last time-interval. After the signal extraction and the signal segmentation, a total of 530 signal segments are generated. The number of room transitions is 21 ± 17 is each time-interval segment, with the duration of the time-interval segments being 2040 ± 290 s. The duration vs. The number of room transitions in each time-interval segment is presented in Figure 9.

In order to assess the frailty status, several features are extracted from the time-interval segments, which assess the mobility of the subject and are designed based on well-known knowledge from other domains of biomedical signal processing involving time-interval signal analysis, and experts’ knowledge in this specific field. The features are presented in Table 3. The frailty status of the subject that the time-interval signal corresponds is included in the feature vector.

The features extracted from the time-interval segments are used for a classification process. The classification dataset includes 530 samples (249 as non-frail, 195 as pre-frail and 84 as frail) and ten features (including annotation). Two separate classification problems were addressed; these are: (i) assessment of the frailty status in the non-frail/pre-frail/frail scale, and (ii) identification of frail subjects, where non-frail and prefrail were considered as a single class. Several well-known classification schemes have been employed in the process: (i) Naïve Bayes classifier (NB), (ii) 10-Nearest Neighbour (kNN), (iii) Neural Networks (NN), (iv) C4.5 Decision Tree (DT), and (v) Random Forests (RF). The ten-fold stratified cross-validation is employed in all classification cases. Results are obtained in terms of average sensitivity, defined as the average of the sensitivity obtained for each class, average positive predictive value (PPV), defined as the average of the positive predictive value obtained for each class, and classification accuracy. The results for the assessment of the frailty status problem are presented in Table 4.

**Figure 10:**
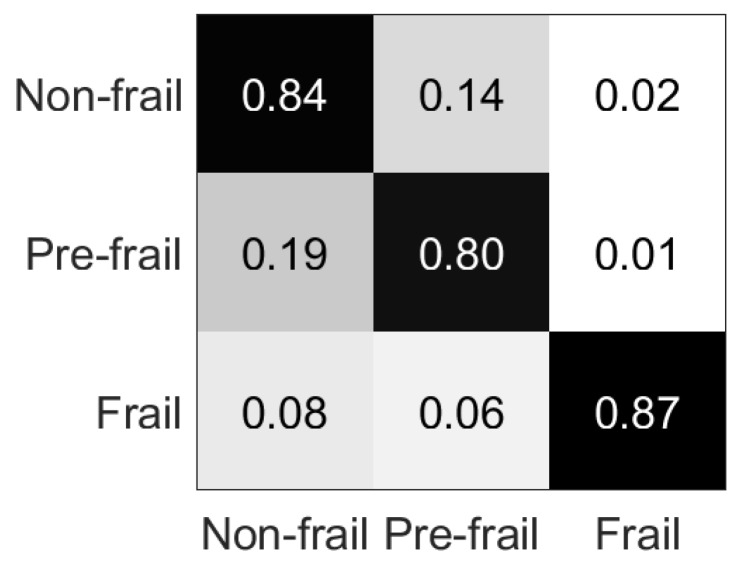
Percentage confusion matrix for Table 5.

The RF classifier obtained the best results, and the respective confusion matrix is presented in Table 5 and Figure 10.

**Figure 11:**
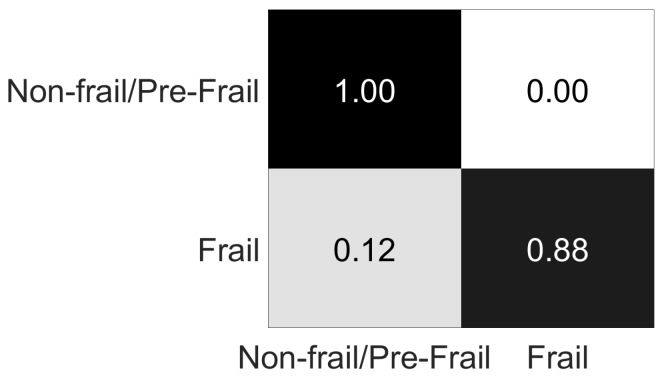
Percentage confusion matrix for Table 7.

The results for the identification of frail subjects problem are presented in Table 6, and the respective confusion matrix for the RF classifier in Table 7 and in Figure 11.

## 5. Discussion

Regarding the evaluation of the system for indoor localization accuracy, two tests were conducted as presented in Figure 7a,b. The increase of the number of used beacons from five to eight shows a small increase in room estimation accuracy from 93.75% to 95.31% while the total number of wrong room estimations was decreased from 12 to 9. The increase in accuracy, was rather expected. However using more beacons the cost increases and the training phase takes more time. As can be seen in Figure 7a,b, the wrong estimation points are located in the limits of the rooms. In all erroneous cases, the error was that the estimated room was a neighboring room to the actual room, e.g., making an estimation of “living room” when the actual room was “bedroom”, or making an estimation of “kitchen” when the actual room was “living room”.

The classification accuracy results for the assessment of the frailty status problem, vary from 27.65% to 82.33% (Table 4). The confusion matrix of the RF classifier reveals that mainly misclassification occurs between non-frail and pre-frail classes (Table 5), with 34 pre-frail cases misclassified as non-frail (being 87.18% of all pre-frail misclassified cases and 38.64% of the overall misclassified cases), and 36 non-frail cases misclassified as pre-frail, (being 83.72% of all non-frail misclassified cases and 40.91% of the overall misclassified cases), while 79.55% of the misclassification occurred between these two classes. This misclassification occurs probably due to similar movement features between these two classes, which seem to be more related than the frail class. This result becomes clearer with the identification of frail subjects problem, where non-frail and pre-frail classes are considered as a single class. The classification accuracy results vary from 42.64% to 97.92% (Table 6), while in the RF classifier (which achieved the best results) only 11 cases are misclassified, as shown in Table 7.

Regarding the classifiers, it is clear that RF outperforms DT and simpler classification techniques (NB, kNN) in both problems. However, NNs achieve significantly lower results compared to RF, in both problems (assessment of the frailty status and identification of frail subjects), and this is probably due to the unbalanced datasets used in both cases, since RF has been reported to handle unbalanced classification datasets more efficiently than NNs [45].

## 6. Conclusions

In this paper, a room-level accuracy indoor localization system is presented for assessing frailty in older people. The system is based on Bluetooth RSSI fingerprints using beacons. It includes two phases, the training phase and the real-time localization phase, which is based on the first one. The focus for the designed system was the ease of installation, the low-cost and the unobtrusiveness. Its accuracy regarding room estimation is above 93%, as tested in realistic conditions.

The proposed localization system was used as a novel approach for the automated assessment of the frailty status of older people. The frailty assessment analysis is based on sequential data, obtained unobtrusively while the subjects were being monitored by the localization system, performing daily activities. The proposed method is based on a low-cost indoor localization system for activity pattern extraction, which focuses on easy installation and limited training by clinical personnel in home environments.The system is based on Bluetooth RSSI fingerprints using beacons. Its accuracy regarding room estimation is above 93%, as tested in realistic conditions. The obtained results indicate that subjects with frailty present distinctive movement patterns, and can be identified with high accuracy (98%), while more granular frailty status can be assessed with acceptable accuracy (>80%).

Future work will focus on obtaining additional data to evaluate the proposed methodology, in particular data from the ACTIVAGE project, the use of other medical information related to the health status of older people and considering different analysis of the time-interval signal. Also, sequence analysis algorithms (such as sequential pattern mining) will be tested for the same classification problems. Future improvements include longer-period monitoring, much longer time frames than 1–7 days, in order to look for changes in trend of individual habits. In addition other smart healthcare services, such as the fall detection system in [46] or [24], which uses accelerometers, could be integrated by the proposed system. These systems in combination with the real-time localization data will increase fall alarm confidence, since certain rooms have higher fall risk than others. Different hardware approaches for the localization system and the use of UWB modules are also included in future work. 

## Figures and Tables

**Table 1:** Room localization systems comparison.

System Reference	Ease of Installation	Accuracy	Cost/Hardware Availability	Suitable for House Environment
**Proposed System**	+++	++	+++	+++
**[19]**	+	++	++	+++
**[20]**	+++	+++	++	+
**[21,22]**	++	+	+	+++

**Table 2:** Frailty status of subjects.

Frailty Status	Total Subjects (Males/Females)
Non-frail	117 (51/66)
Pre-frail	131 (42/89)
Frail	23 (9/14)

**Table 3:** Description of the features extracted from each time-interval segment.

Feature	Description
Number of room transitions	The number of total room transitions in the time interval signal
Room transition average time duration	The average of the time intervals in the time interval signal
Room transition standard deviation of time duration	The standard deviation of the time intervals in the time interval signal
Number of fast room transitions	Number of time inervals with duration <= 15 s
Number of slow room transitions	Number of time inervals with duration > 600 s
Percentage of fast room transitions	Ratio of the number of fast room transitions to the number of room transitions
Percentage of slow room transitions	Ratio of the number of slow room transitions to the number of room transitions
Normalised number of fast room transitions	$\frac{\mathrm{Number}\;\mathrm{of}\;\mathrm{fast}\;\mathrm{room}\;\mathrm{transitions}}{(\mathrm{Number}\;\mathrm{of}\;\mathrm{rooms})*(\mathrm{Number}\;\mathrm{of}\;\mathrm{room}\;\mathrm{transitions})}$
Normalised number of slow room transitions	$\frac{\mathrm{Number}\;\mathrm{of}\;\mathrm{slow}\;\mathrm{room}\;\mathrm{transitions}}{(\mathrm{Number}\;\mathrm{of}\;\mathrm{rooms})*(\mathrm{Number}\;\mathrm{of}\;\mathrm{room}\;\mathrm{transitions})}$

**Table 4:** Results for the assessment of the frailty status problem.

Classifier	Average Sensitivity	Average PPV	Classification Accuracy
**NB**	36.13%	37.97%	27.65%
**kNN**	54.20%	52.63%	57.01%
**NN**	44.37%	43.57%	55.11%
**DT**	70.83%	72.17%	71.78%
**RF**	83.83%	85.20%	82.33%

**Table 5:** Confusion matrix for the RF classifier for the assessment of the frailty status problem.

		Classified		
		**Non-Frail**	**Pre-Frail**	**Frail**
**Dataset**	**Non-Frail**	206	36	7
	**Pre-Frail**	34	156	5
	**Frail**	4	2	78

**Table 6:** Results for the identification of frail subjects problem.

Classifier	Average Sensitivity	Average PPV	Classification Accuracy
**NB**	52.50%	54.30%	42.64%
**kNN**	52.70%	50.70%	82.08%
**NN**	42.05%	49.80%	83.77%
**DT**	78.20%	80.15%	88.49%
**RF**	94.20%	98.75%	97.92%

**Table 7:** Confusion matrix for the RF classifier for the identification of frail subjects problem.

		Classified	
		Non-Frail/Pre-Frail	Frail
**Dataset**	**Non-frail/Pre-frail**	435	11
	**Frail**	0	84
